# cAMP response element binding protein (CREB) activates transcription via two distinct genetic elements of the human glucose-6-phosphatase gene

**DOI:** 10.1186/1471-2199-6-2

**Published:** 2005-01-19

**Authors:** Gerald Thiel, Jude Al Sarraj, Luisa Stefano

**Affiliations:** 1 Department of Medical Biochemistry and Molecular Biology, Building 44, University of Saarland Medical Center, D-66421 Homburg, Germany

## Abstract

**Background:**

The enzyme glucose-6-phosphatase catalyzes the dephosphorylation of glucose-6-phosphatase to glucose, the final step in the gluconeogenic and glycogenolytic pathways. Expression of the glucose-6-phosphatase gene is induced by glucocorticoids and elevated levels of intracellular cAMP. The effect of cAMP in regulating glucose-6-phosphatase gene transcription was corroborated by the identification of two genetic motifs CRE1 and CRE2 in the human and murine glucose-6-phosphatase gene promoter that resemble cAMP response elements (CRE).

**Results:**

The cAMP response element is a point of convergence for many extracellular and intracellular signals, including cAMP, calcium, and neurotrophins. The major CRE binding protein CREB, a member of the basic region leucine zipper (bZIP) family of transcription factors, requires phosphorylation to become a biologically active transcriptional activator. Since unphosphorylated CREB is transcriptionally silent simple overexpression studies cannot be performed to test the biological role of CRE-like sequences of the glucose-6-phosphatase gene. The use of a constitutively active CREB2/CREB fusion protein allowed us to uncouple the investigation of target genes of CREB from the variety of signaling pathways that lead to an activation of CREB. Here, we show that this constitutively active CREB2/CREB fusion protein strikingly enhanced reporter gene transcription mediated by either CRE1 or CRE2 derived from the glucose-6-phosphatase gene. Likewise, reporter gene transcription was enhanced following expression of the catalytic subunit of cAMP-dependent protein kinase (PKA) in the nucleus of transfected cells. In contrast, activating transcription factor 2 (ATF2), known to compete with CREB for binding to the canonical CRE sequence 5'-TGACGTCA-3', did not transactivate reporter genes containing CRE1, CRE2, or both CREs derived from the glucose-6-phosphatase gene.

**Conclusions:**

Using a constitutively active CREB2/CREB fusion protein and a mutant of the PKA catalytic subunit that is targeted to the nucleus, we have shown that the glucose-6-phosphatase gene has two distinct genetic elements that function as *bona fide *CRE. This study further shows that the expression vectors encoding C2/CREB and catalytic subunit of PKA are valuable tools for the study of CREB-mediated gene transcription and the biological functions of CREB.

## Background

The glucose-6-phosphatase system consists of the glucose-6-phosphate catalytic subunit (EC 3.1.3.9), embedded in the membrane of the endoplasmic reticulum (ER) via nine transmembrane domains, and the membrane spanning translocases, responsible in carrying either the substrate into the ER or the product from the ER [1]. Transport of substrate and product is necessary due to the orientation of the active site of the glucose-6-phosphatase enzyme towards the luminal side of the ER. Glucose-6-phosphate, the end product of both gluconeogenesis and glycogenolysis in the liver, is hydrolyzed by the glucose-6-phosphatase system allowing the liberation of glucose into the circulation. Thus, glucose-6-phosphatase plays a key role in the homeostasis of blood glucose. Mutations in the gene encoding the catalytic subunit of glucose-6-phosphatase are responsible for the development of glycogen storage disease type 1, also known as Gierke disease [2]. In animal models of diabetes mellitus, glucose-6-phosphatase activity is increased along with mRNA and protein levels [3]. Accordingly, inhibitors of glucose-6-phosphatase or the G6PT transporter are used for the treatment of type 2 diabetes.

The glucose-6-phosphatase encoding gene is regulated by a variety of extracellular signaling molecules, including glucose, insulin, glucocorticoids, and cAMP. Insulin decreases the level of glucose-6-phosphatase mRNA in the liver and in hepatoma cells and three functionally distinct insulin response elements have been identified [4]. Glucocorticoids elevate glucose-6-phosphatase promoter activity mediated by a glucocorticoid response element and hepatocyte nuclear factor 1 [5]. Conflicting results were published concerning the stimulation of glucose-6-phosphatase promoter activity by elevated intracellular cAMP concentrations. While dibutyryl cAMP alone did not significantly stimulate transcription of a luciferase reporter gene under control of 1.2 kb of the human glucose-6-phosphatase promoter in H4IIIE hepatoma cells [6], a later report by the same group described a ≈ 2-fold stimulation of glucose-6-phosphatase promoter activity with dibutyryl cAMP [7]. The sequence from -161 to -152 of the human glucose-6-phosphatase promoter, including the motif 5'-TTTACGTAA-3', was proposed to mediate the effect of dibutyryl cAMP on reporter gene transcription [7]. In HepG2 cells a glucose-6-phosphatase promoter/chloramphenicol acetyltransferase reporter gene showed a 2 to 3-fold enhancement in transcription following stimulation of the cells with dibutyryl cAMP [8]. Here, the sequence from -136 to -129 of the human glucose-6-phosphatase gene, including the motif 5'-TTGCATCAA-3', was proposed to be essential to couple dibutyryl cAMP stimulation with enhanced reporter gene transcription [8].

The most important and best characterized protein that connects an elevation of intracellular cAMP concentrations with enhanced transcription of selected genes is the CRE binding protein CREB, a basic region leucine zipper protein. CREB plays an essential role in the regulation of glucose-6-phosphatase gene transcription in the liver, as exemplified by the fact that transgenic mice expressing a dominant-negative CREB mutant in the liver show reduced mRNA levels of glucose-6-phosphatase [9]. CREB not only mediates stimulus-transcription coupling of the cAMP signaling pathway but functions as a point of convergence of many other signaling molecules involving calcium, neurotrophins, tumor promoters, and growth factors [10]. CREB is inactive in the dephosphorylated state and turns into an activator upon phosphorylation. The key enzyme leading to CREB activation is the cAMP-dependent protein kinase (PKA), but CREB serves also as a substrate for calcium/calmodulin-dependent protein kinase IV and the mitogen-and stress-activated kinases MSK1 and 2 [11,12]. To study CREB regulated gene transcription, a signaling cascade needs to be activated that leads to phosphorylation and activation of CREB. This can be accomplished by adding cAMP analogues such as dibutyryl cAMP that passes the plasma membrane or via a direct activation of adenylate cyclase by forskolin. This experimental setting is problematic in that high levels of phosphodiesterase enzymes in the cells may immediately hydrolyze cAMP, with the result that no cAMP-mediated gene transcription can be monitored [13]. Moreover, elevated levels of cAMP may additionally activate the EPAC/Rap1 pathway, making it somewhat difficult to attribute the effect of cAMP solely to the cAMP/PKA signaling pathway. Many functions of cAMP previously attributed to PKA may be in fact the result of EPAC activation [14].

Instead of increasing the intracellular levels of cAMP some investigators employed an overexpression strategy using the catalytic subunit of cAMP-dependent protein kinase to analyze cAMP regulated genes. This approach has the advantage that by excluding a parallel signaling cascade via the cAMP-inducible nucleotide exchange factor EPAC, only the biological outcome of cAMP-dependent protein kinase activation is studied. However, the translocation of the catalytic subunit of cAMP-dependent protein kinase into the nucleus is not very efficient and may rely solely on diffusion [15]. Thus high amounts of expression vector in the range of 1 to 5 μg/plate are often transfected [16-18] in order to observe an effect on gene transcription. Naturally, these high amounts of expressed catalytic subunit are far away from the physiological concentrations within the cells.

Here, we report on the regulation of glucose-6-phosphatase gene transcription by CREB and PKA. Using a constitutively active CREB2/CREB fusion protein, that does not require phosphorylation for activation, and a nuclear-targeted mutant of the catalytic subunit of PKA, we show that CREB activates transcription of glucose-6-phosphatase promoter/luciferase reporter genes via two distinct genetic elements. In contrast, the bZIP protein ATF2, known to compete with CREB for binding to the canonical CRE, did not transactivate these reporter genes containing CRE1, CRE2, or both CREs derived from the glucose-6-phosphatase gene.

## Results

### The proximal region of the human glucose-6-phosphatase gene contains two cyclic AMP response element like motifs

**Figure 1:**
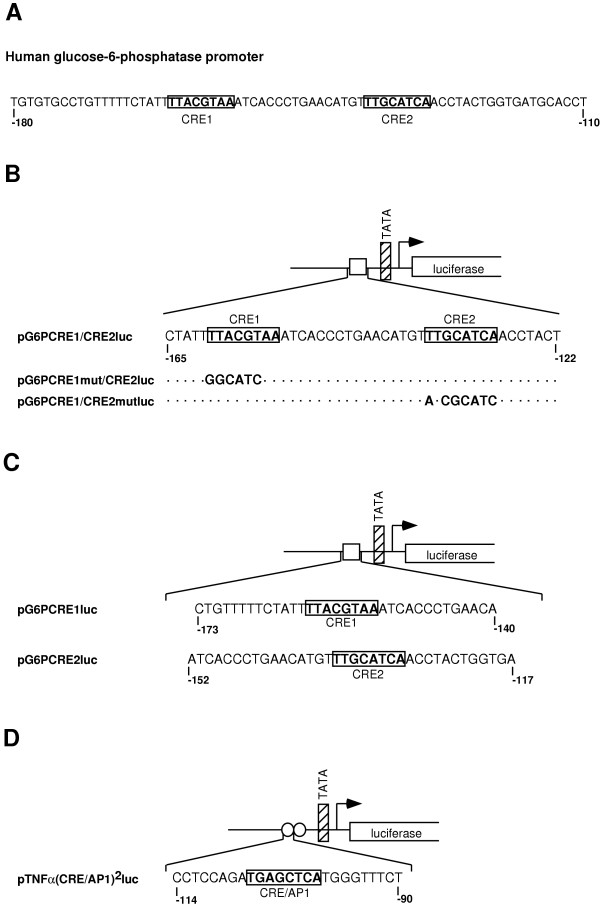
**The human glucose-6-phosphatase gene promoter**. (A) Sequence of a portion of the human glucose-6-phosphatase gene promoter including the CRE-like sequences CRE1 and CRE2. (B) The reporter plasmid pG6PCRE1/CRE2luc contains a minimal promoter consisting of the human immunodeficiency virus TATA box, the adenovirus major late promoter initiator element, the luciferase open reading frame, and glucose-6-phosphatase promoter sequences encompassing both CRE-like sequences CRE1 and CRE2. The reporter plasmids pG6PCRE1mut/CRE2luc and pG6PCRE1/CRE2mutluc carry mutations in either the CRE1 or the CRE2, to inactivate these sites. (C) Reporter plasmids pG6PCRE1luc and pG6PCRE2luc contain glucose-6-phosphatase promoter sequences encompassing either the CRE1 or the CRE2. (D) The reporter plasmid pTNFα(CRE/AP1)^2^luc contains two identical copies of the composite CRE/AP1 sequence derived from the human TNFα gene, upstream of a minimal promoter. The sequence of one of these motifs is depicted.

Fig. 1A shows part of the proximal region of the human glucose-6-phosphatase gene. As indicated, two CRE-like sequences are present, resembling the canonical CRE sequence 5'-TGACGTCA-3'. The distal CRE-like site (CRE1) and the proximal CRE-like site (CRE2) differ on two or three positions, respectively, in comparison to the canonical CRE. To investigate the biological function of these elements we constructed a battery of reporter plasmids containing both CRE-like elements or only one of them (Fig. 1B,C). Mutations were introduced into CRE1 and CRE2 sites to prevent CREB binding [19]. As a control, we generated a reporter plasmid containing two copies of a CRE/AP1-like element derived from the human tumor necrosis factor (TNF) α gene promoter (Fig. 1D). The TNFα gene belongs to the target genes of CREB [10]. Additionally, the reporter genes contained a TATA box derived from the HIV long terminal repeat, an initiator element from the adenovirus major late promoter and the luciferase open reading frame.

### A constitutively active CREB2/CREB fusion protein transactivates reporter genes with either CRE1 or CRE2 of the glucose-6-phosphatase gene in their regulatory regions

**Figure 2:**
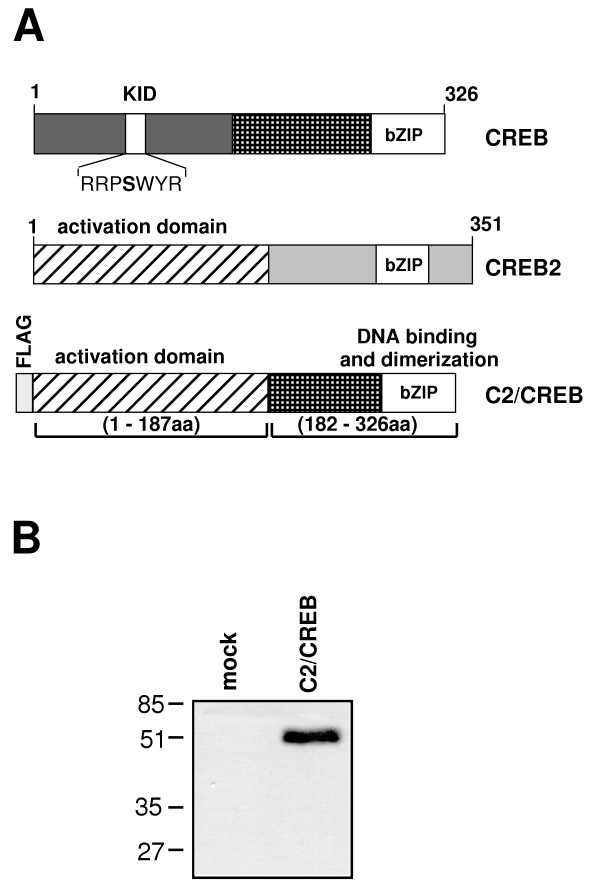
**Schematic representation and expression of the constitutively active CREB2/CREB fusion protein C2/CREB**. (A) Schematic representation of the modular structure of CREB, CREB2, and C2/CREB. The basic region leucine zipper domain (bZIP) is indicated. The chimeric bZIP protein C2/CREB consists of the constitutively active transcriptional activation domain of CREB2 and the bZIP domain of CREB, responsible for dimerization and DNA-binding. (B) Western Blot analysis of HepG2 cells transfected with an expression vector encoding C2/CREB. As a control, extracts from mock transfected HepG2 cells were analyzed. Western blots were probed with an antibody against the FLAG-tag.

The fact that unphosphorylated CREB is transcriptionally silent excludes a simple overexpression strategy to analyze the biological impact of the CRE-like sequences within the glucose-6-phosphatase gene. The use of natural inducers of CREB such as glucagon or epinephrine, that all elevate the cAMP concentration in the cells, also triggers pleiotropic responses by activating the cAMP-dependent protein kinase and the EPAC/Rap1 pathway, so that the biological outcome cannot be attributed solely to CREB activation. We therefore designed a constitutively active CREB2/CREB fusion protein to uncouple the investigation of glucose-6-phosphatase gene transcription from signaling pathways in the cell. Fig. 2A shows the structural domains of the basic region leucine zipper (bZIP) transcription factors CREB and CREB2. Both proteins contain a bZIP domain on their C-termini responsible for dimerization and DNA-binding. The N-termini of CREB and CREB2 contain activation domains. While the activation domain of CREB2 is constitutively active and transferable to heterologous DNA-binding domains [20], the major activation domain of CREB is controlled by phosphorylation. We expressed the bZIP domain of CREB, which is responsible for DNA binding and dimerization, as a fusion protein with the constitutively active transcriptional activation domain of CREB2, generating the chimeric transcription factor C2/CREB (Fig. 2A). The C2/CREB fusion protein contains additionally an immunological tag used for the detection of the protein. Proteins derived from nuclear extracts of HepG2 human hepatoma cells (mock) or HepG2 cells transfected with an expression vector encoding C2/CREB were fractionated by SDS-PAGE. The fusion protein was identified by Western Blot analysis using antibodies targeting the FLAG epitope. Fig. 2B shows that the CREB2/CREB fusion protein was synthesized as expected.

**Figure 3:**
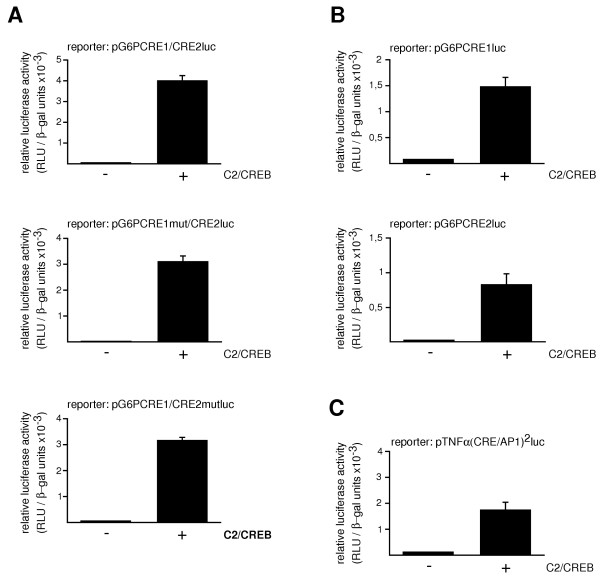
**Biological activity of C2/CREB, a constitutively active CREB2/CREB fusion protein**. One of the reporter plasmids pG6PCRE1/CRE2luc, pG6PCRE1mut/CRE2luc pG6PCRE1/CRE2mutluc (A), pG6PCRE1luc, pG6PCRE2luc (B), or pTNFα(CRE/AP1)^2^luc (C) (1 μg/plate) was transfected into HepG2 cells together with the pRSVβ internal standard plasmid (2 μg/plate), encoding β-galactosidase under the control of the Rous sarcoma virus long terminal repeat, and either the "empty" expression vector pCMV5 or an expression vector encoding C2/CREB (20 ng plasmid/plate). The data are presented as the ratio of luciferase activity (light units) to β-galactosidase units (OD units) measured in the cell extracts. The mean +/- SD is depicted.

To study the regulation of the glucose-6-phosphatase promoter/luciferase reporter genes by C2/CREB, we decided to use the human hepatoma cell line HepG2, as it has been reported that activation of the cAMP signaling pathway stimulates glucose-6-phosphatase gene transcription in these cells [17,21,22]. HepG2 cells were transfected with one of the luciferase reporter plasmids pG6PCRE1/CRE2luc, pG6PCRE1mut/CRE2luc, pG6PCRE1/CRE2mutluc, pG6PCRE1luc or pG6PCRE2luc together with either the "empty" expression vector pCMV5 (denoted "-") or an expression vector encoding C2/CREB (plasmid pCMV-FLAG-C2/CREB) (denoted "+"). As a control, we transfected the pTNFα(CRE/AP1)^2^luc reporter plasmid that contains two copies of the composite CRE/AP1 element of the TNFα promoter. We transfected additionally plasmid pRSVβ, encoding β-galactosidase under the control of the Rous sarcoma virus long-terminal repeat, to correct for variations in transfection efficiencies. Luciferase activities were normalized for transfection efficiency by dividing luciferase light units by β-galactosidase activities. The results of the transfection experiments are depicted in Fig. 3. Transcription of the pG6PCRE1/CRE2luc reporter gene, that contains both CRE-like sequences in its regulatory region, was strongly induced following expression of C2/CREB (Fig. 3A, upper panel). Mutation of CRE1 or CRE2 did not abolish the transactivation potential of C2/CREB (Fig. 3A, middle, bottom panel), indicating that both CRE-like sequences function independently as *bona fide *CREs. This conclusion was confirmed by transfection experiments of reporter plasmids having only one of the CRE-like sequences in the regulatory regions. Fig. 3B shows that both reporter genes pG6PCRE1luc and pG6PCRE2luc were transactivated by C2/CREB. Finally, C2/CREB also transactivated the pTNFα(CRE/AP1)^2^luc reporter gene via the CRE/AP1 element (Fig. 3C). We conclude that both CRE1 and CRE2 motifs of the glucose-6-phosphatase gene promoter function as *bona fide *CREs.

### Expression of a nuclear-targeted catalytic subunit of cAMP-dependent protein kinase

**Figure 4:**
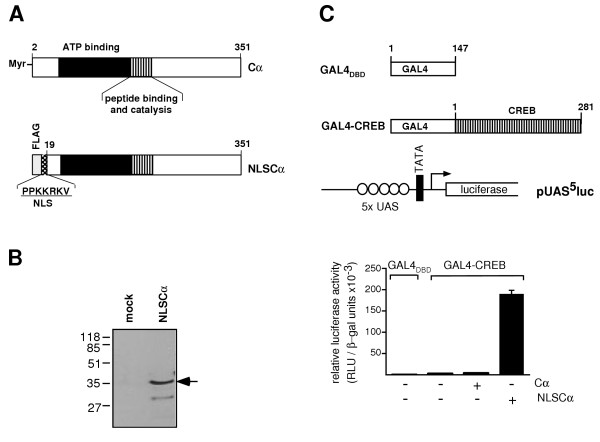
**Schematic representation and biological activity of the nuclear targeted mutant of the catalytic subunit of cAMP-dependent protein kinase**. (A) Schematic representation of the modular structure of the catalytic subunit of cAMP-dependent protein kinase (Cα) and the mutant NLSCα. The wild-type enzyme is myristylated as indicated (Myr). The sequence of the nuclear localization signal derived from the SV40 large T antigen and the triple FLAG epitope in NLSCα are shown. (B) Western Blot analysis of HepG2 cells transfected with an expression vector encoding NLSCα. As a control, extracts from mock transfected HepG2 cells were analyzed. Western blots were probed with an antibody against the FLAG-tag. (C) Modular structure of the GAL4-CREB fusion protein. The protein consists of the DNA-binding domain of GAL4 (amino acids 1–147) and the activation domain of CREB (amino acids 1–281). The reporter plasmid pUAS^5^luc contains a transcription unit encompassing the luciferase open reading frame and a minimal promoter that consists of five copies of the upstream activating sequence (UAS), a TATA box derived from the HIV long terminal repeat and the initiator element from the adenovirus major late promoter. The reporter plasmid pUAS^5^luc (1 μg/plate) was transfected into HepG2 cells together with the pRSVβ internal standard plasmid (2 μg/plate) and either an expression vector encoding the DNA binding domain of GAL4 (plasmid pM1) or the GAL4-CREB fusion protein (1 μg plasmid/plate). In addition, cells were transfected with an expression vector encoding either the wild-type (Cα) or mutated (NLSCα) form of cAMP-dependent protein kinase (100 ng/plate). Forty-eight hours post-transfection cell extracts were prepared and the β-galactosidase and luciferase activities of these extracts were determined.

The structural domains of the catalytic subunit of cAMP-dependent protein kinase (ATP:protein phosphotransferase, EC 2.7.1.37) are depicted in Fig. 4A. The protein is myristylated on the N-terminus, but myristylation is nonessential for enzyme activation [23]. We modified the N- terminal region of the protein by adding a nuclear targeting signal derived from the SV40 large T antigen (NLS) and a FLAG epitope to facilitate immunological detection. This mutant termed NLSCα should be sorted to the nuclear compartment, due to the presence of the NLS. HepG2 cells were transfected with an expression vector encoding NLSCα. Nuclear extracts of these cells were fractionated by SDS-PAGE and analyzed by Western blotting. Fig. 4B shows that the modified catalytic subunit of cAMP-dependent protein kinase was synthesized as expected.

To test the biological activity of NLSCα, in comparison to the wild-type form of the catalytic subunit (Cα), we measured the activity of the phosphorylation-regulated transcription factor CREB using a fusion protein consisting of the GAL4 DNA-binding domain fused to the kinase inducible activation domain of CREB. The modular structure of the GAL4-CREB fusion protein is depicted in Fig. 4C. Transcriptional activation was monitored by co-transfection of the reporter plasmid pUAS^5^luc that contains five copies of the GAL4 binding site termed "upstream activating sequence" (UAS) upstream of a luciferase reporter gene (Fig. 4C). Since mammalian cells do not express transcription factors that bind to the UAS, this system directly measures the effect of NLSCα or Cα on the transcriptional activation potential of CREB. The reporter plasmid pUAS^5^luc and the expression plasmid that encodes GAL4-CREB were transfected into HepG2 cells together with either an "empty" expression vector (denoted "-") or expression vectors encoding Cα or NLSCα. Transfection efficiency was monitored by co-transfecting pRSVβ. As a control, plasmid pM1 encoding only the DNA binding domain of GAL4 (GAL4_DBD_) was transfected. Cell extracts were prepared forty-eight hours later and β-galactosidase and luciferase activities were determined. Fig. 4C shows that the modified form of PKA catalytic subunit (NLSCα) was highly potent in stimulating the transcriptional activation potential of CREB. The activation was on the order of 60-fold. In contrast, no transcriptional activation was observed following overexpression of the wild-type form of the catalytic subunit.

**Figure 5:**
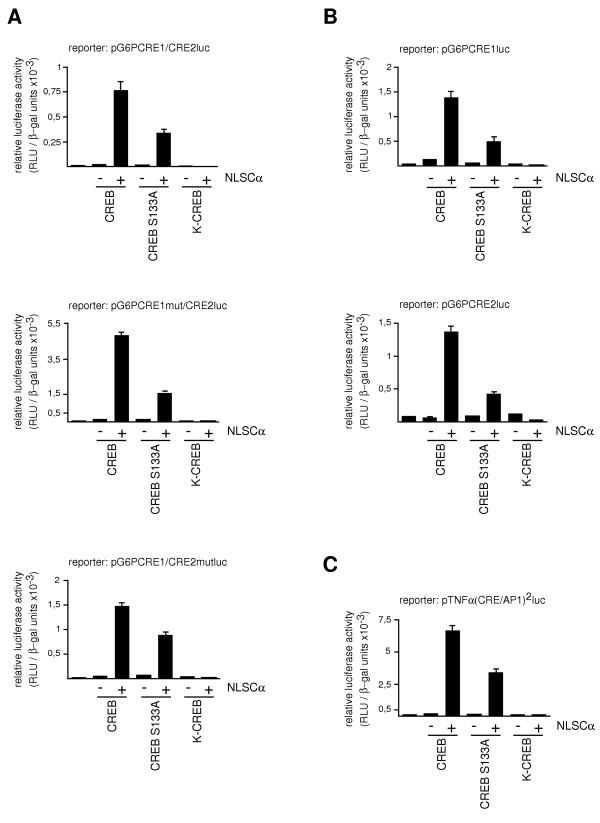
**Transcriptional activity of CREB and CREB mutants in the presence of a nuclear targeted mutant of the catalytic subunit of cAMP-dependent protein kinase**. One of the reporter plasmids pG6PCRE1/CRE2luc, pG6PCRE1mut/CRE2luc pG6PCRE1/CRE2mutluc (A), pG6PCRE1luc, pG6PCRE2luc (B), or pTNFα(CRE/AP1)^2^luc (C) (1 μg/plate) was transfected into HepG2 cells together with the pRSVβ internal standard plasmid (2 μg/plate) and the "empty" expression vector pCMV5 or an expression vector encoding either CREB, CREBS133A, or K-CREB (20 ng plasmid/plate). In addition, an expression vector encoding NLSCα (100 ng/plate) was transfected. Forty-eight hours post-transfection cell extracts were prepared and the β-galactosidase and luciferase activities of these extracts were determined. The data are presented as the ratio of luciferase activity (light units) to β-galactosidase units (OD units) measured in the cell extracts. The mean +/- SD is depicted.

Next, we tested the biological activity of NLSCα with regard to transcription of the glucose-6-phosphatase promoter/luciferase reporter genes. In the experiments, we additionally overexpressed wild-type CREB, the CREBS133A containing a serine to alanine mutation at position 133, and K-CREB, a CREB mutant containing the point mutation R286L within the basic DNA-binding domain, impairing DNA-binding [24]. Transfection of expression vectors encoding the wild-type form of CREB, CREBS133A, or K-CREB did not significantly change basal transcription of the reporter genes. However, transfection of an NLSCα expression vector strongly stimulated transcription of the glucose-6-phosphatase promoter/luciferase reporter genes, containing both or one of the CRE-like sequences of the glucose-6-phosphatase promoter (Fig. 5A,B). These results confirm the previous observations, obtained via overexpression of C2/CREB, that both CRE motifs of the glucose-6-phosphatase gene function as independent genetic elements responsive to couple elevated cAMP and PKA levels with enhanced glucose-6-phosphatase gene transcription. Similarly, coexpression of wild-type CREB and NLSCα stimulated reporter gene transcription mediated by the CRE/AP1 element derived from the TNFα gene (Fig. 5C). Surprisingly, we still detected reporter gene activation following coexpression of NLSCα with CREBS133A, lacking the major PKA phosphorylation site. Compared to the coexpression experiments of NLSCα with the wild-type form of CREB, the reduced activation of reporter gene transcription in coexpression experiments of NLSCα with CREBS133A indicates that NLSCα catalyzed phosphorylation of serine residue 133 of CREB is important for reporter gene transcription. The fact that CREBS133A is still able to transactivate the reporter genes following expression of NLSCα suggests that NLSCα triggers further phosphorylation reactions leading to enhanced transcription via the CRE-like sequences within the glucose-6-phosphatase gene. In contrast, expression of K-CREB is transcriptionally inactive, in the presence or absence of NLSCα, indicating that DNA-binding is a prerequisite for CREB and CREBS133A to transactivate the reporter genes.

### Biological activity of a constitutively active CREB2/ATF2 fusion protein on glucose-6-phosphatase promoter/luciferase reporter genes

**Figure 6:**
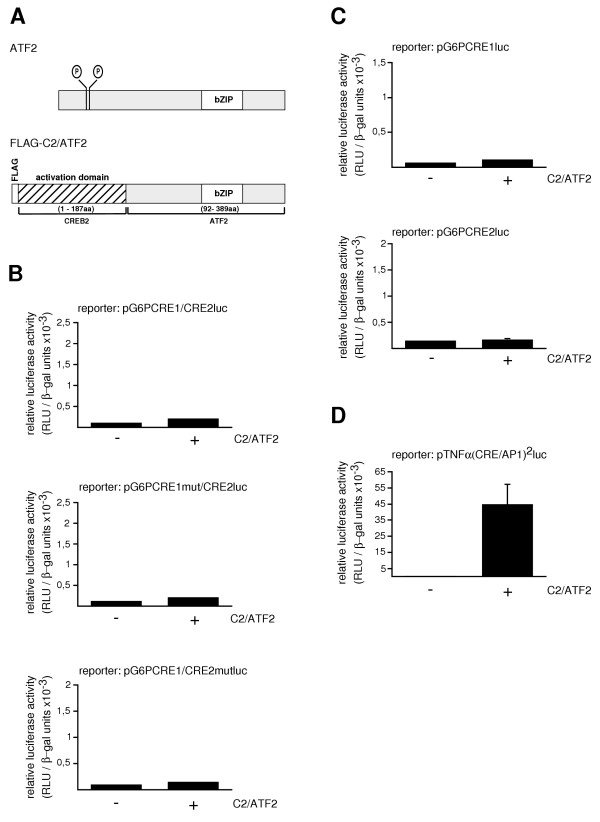
**Biological activity of constitutively active ATF2 fusion protein towards glucose-6-phosphatase promoter-containing reporter genes**. (A) Schematic representation of the modular structure of C2/ATF2. This chimeric bZIP protein consists of the constitutively active transcriptional activation domain of CREB2 and the bZIP domain of ATF2, responsible for dimerization and DNA-binding. (B, C, D) HepG2 cells were transfected with one of the reporter plasmids pG6PCRE1/CRE2luc, pG6PCRE1mut/CRE2luc pG6PCRE1/CRE2mutluc (B), pG6PCRE1luc, pG6PCRE2luc (C), or pTNFα(CRE/AP1)^2^luc (D), the pRSVβ internal reference plasmid, and either the "empty" expression vector pCMV5 or an expression vector encoding C2/ATF2 (100 ng expression plasmid/plate). Lysates were prepared forty-eight hours post-transfection and β-galactosidase and luciferase activities were measured. The mean +/- SD is depicted.

The transcription factor ATF2, a substrate of stress-activated protein kinases, has been reported to bind to the classical cAMP responsive element (CRE) 5'-TGACGTCA-3' [25]. Recently, we confirmed this observation showing that ATF2 and CREB compete for binding to the CRE of the secretogranin II gene [26]. ATF2 was proposed to also bind with high affinity to the related DNA target sequence 5'-TTACGTAA-3' [19], which is identical to the CRE1 of the glucose-6-phosphatase gene promoter. We tested the biological activity of ATF2 on reporter genes containing sequences of the glucose-6-phosphatase promoter region. As a control, we used a reporter gene containing two copies of the CRE/AP-1 element of the TNFα gene. ATF2 has been shown to stimulate TNFα promoter activity [27]. The unphosphorylated form of ATF2 is transcriptionally inactive, due to an inhibitory intramolecular interaction between the activation domain of ATF2 and the bZIP domain of ATF2 [28]. We therefore expressed a constitutively active CREB2/ATF2 fusion protein to investigate whether ATF2 functions as a transactivator for reporter genes containing the CRE1 and/or CRE2 sequences of the glucose-6-phosphatase gene in the regulatory region. The domain structure of this C2/ATF2 fusion protein is depicted in Fig. 6A. HepG2 cells were transfected with one of the reporter plasmids (pG6PCRE1/CRE2luc, pG6PCRE1mut/CRE2luc, pG6PCRE1/CRE2mutluc, pG6PCRE1luc, and pG6PCRE2luc), the internal standard plasmid pRSVβ, and an expression vector encoding C2/ATF2. As a control, we analyzed the reporter plasmid pTNFα(CRE/AP1)^2^luc. The results show that C2/ATF2 was unable to transactivate the reporter genes containing CRE1, CRE2, or both CRE-like sequences derived from the glucose-6-phosphatase gene in the regulatory region, indicating that glucose-6-phosphatase gene transcription is not regulated by ATF2 via the two CRE-like sequences. In contrast, C2/ATF2 strongly activated transcription of the pTNFα(CRE/AP1)^2^luc reporter gene, confirming that TNFα gene transcription is regulated by ATF2.

## Discussion

The gene encoding glucose-6-phosphatase is regulated by increased levels of intracellular cAMP, but the regulatory sites responsible for cAMP-induced gene transcription are still a matter of controversy. The objective of this study was to characterize the genetic elements that function as *cis*-acting sites for transactivation by CREB and that also respond to activated PKA in the nucleus. Two distinct genetic elements had been suggested to couple enhanced levels of cAMP and elevated PKA activity with increased glucose-6-phosphatase gene transcription [8,17] and we intended to clarify which of these elements is required for transactivation of the glucose-6-phosphatase gene by CREB.

The reason for the differences in the published reports concerning cAMP-regulated glucose-6-phosphatase gene transcription can be explained by the technical problems that occurs following elevation of the intracellular cAMP concentration, as outlined in the introduction section. To solve these problems, we prefered to measure "transcriptional activation" instead of "transcription factor/DNA-binding" because although DNA-binding is required for a subsequent transcriptional activation by CREB, an enhanced binding activity of a transcription factor to DNA, monitored by an *in vitro *binding assay, does not necessarily prove an enhanced transcriptional activation potential of this protein [29]. In addition, we expressed a constitutively active CREB2/CREB fusion protein that was highly active in transactivating CREB-responsive target genes. Using this strategy we avoided the use of dibutyryl cAMP or forskolin, that may trigger other biological responses. Furthermore, only nanomolar levels of the expression vector encoding C2/CREB were required to show that both CRE-like sequences (CRE1 and CRE2) within the glucose-6-phosphatase gene function as target sites for an active CREB. Thus, both the reported genetic elements, the sequence from -161 to -152 [7] and the sequence from -136 to -129 [8] of the human glucose-6-phosphatase promoter, mediated reporter gene transactivation by CREB. The fact that the glucose-6-phosphatase gene contains two distinct genetic elements for the regulation by CREB is not surprising as multiple CREs have been found in other genes encoding for instance ICER, MKP-1, Nur77 or Egr-4 [30].

Using an overexpression strategy for the catalytic subunit of PKA, it has been reported that PKA directly stimulates reporter gene transcription under control of CRE1, whereas CRE2 was unable to connect elevated PKA activity with enhanced CRE2-controlled reporter gene transcription [17]. The authors concluded that only the CRE1, but not the CRE2 functions as *bona fide *CRE [17]. In this study a concentration of 5 μg of expression vector encoding the catalytic subunit of PKA was used. In the past, we also employed this overexpression strategy for the catalytic subunit of PKA in the laboratory [29,31] and observed that high levels of expression vectors are required to monitor an effect on gene transcription. We also observed that transcription of the reference gene encoding β-galactosidase under control of the Rous sarcoma virus long terminal repeat was changed following overexpression of the catalytic subunit, although no CRE has been mapped within this promoter/enhancer region. This fact indicates that transfection of micromolar levels of expression vectors encoding the catalytic subunit of PKA disturbs the machinery of transcription and the data obtained by this approach may not always depict the real picture. Likewise, it has been known for many years that expression of the strong transcriptional activator VP16 induces transcriptional repression within the cells, due to squelching. In the study reported here, expression of a modified catalytic subunit that had a nuclear targeting signal, activated reporter gene transcription very efficiently following transfection of nanomolar amounts of the expression vector. In fact, we used a 50-fold lower amount of expression vector compared to the study by Streeper et al. [17]. Transfection of nanomolar concentrations of an expression plasmid encoding the wild-type catalytic subunit of PKA did not show any effect on reporter gene transcription. These experiments, involving an overexpression of NLSCα, showed an enhanced transcription of reporter genes having either an intact CRE1 or CRE2 in its regulatory region, indicating that both CRE1 and CRE2 functioned as *bona fide *CRE. We also observed an activation of glucose-6-phosphatase promoter/luciferase reporter gene transcription following coexpression of CREBS133A with NLSCα. Phosphorylation of the serine 133 residue of CREB is the predominant mechanism to enhance the transcriptional activation potential of CREB via recruitment of the coactivator CREB binding protein (CBP) and its paralogue p300 to the promoter. Accordingly, we observed a clear reduction of reporter gene transcription in coexpression studies of NLSCα together with CREBS133A instead of wild-type CREB. However, expression of CREBS133A still contributed to reporter gene transcription in the presence of NLSCα, suggesting that either other residues of CREBS133A or other promoter-bound proteins involved in the regulation of CREB-mediated transcription are phosphorylated. PKA can phosphorylate CBP directly or other components of the transcriptional machinery downstream from CREB [32,33]. Nonetheless, the experiments confirmed that both CRE-like sequences within the glucose-6-phosphatase gene function as genetic elements to mediate transactivation via CREB.

In contrast to the expression experiments involving C2/CREB, we did not detect an effect of C2/ATF2, a constitutively active CREB2/ATF2 fusion protein, on glucose-6-phosphatase promoter/luciferase reporter gene transcription, despite the fact that the sequence 5'-TTACGTAA-3', which is identical to the CRE1 site of the glucose-6-phosphatase promoter, has been shown to function as a high affinity binding site for ATF2 in vitro [19]. This discrepancy supports the view that transcriptional bioassays and not in vitro DNA-protein binding experiments describe the biological activities of transcription factors. The CRE-like sequences CRE1 and CRE2 of the glucose-6-phosphatase gene are therefore strikingly different from the CRE/AP1 site of the TNFα gene, that is strongly activated by C2/ATF2. Recently, we showed that ATF2 is able to specifically transactivate CRE-containing genes and we identified the secretogranin II gene as a target gene for ATF2 [26]. We also showed that C2/ATF2 only marginally enhanced transcription of a reporter gene carrying four copies of the c-Fos CRE in its regulatory region, while transcription regulated by the tyrosine hydroxylase promoter was not upregulated at all. Thus, ATF2 distinguishs between different CRE-containing genes. The biological activity of ATF2 is regulated by stress-activated protein kinases and ATF2 is thought to play an important role in the cellular stress response. Our data sheds light on the fact that expression of glucose-6-phosphatase is not connected – via ATF2 – to the cellular stress response, in contrast to the TNFα gene.

## Conclusions

We have shown that there are two distinct CREB-responsive sites in the glucose-6-phosphatase gene promoter that are responding to either a constitutively active CREB or elevated concentrations of the catalytic subunit of cAMP-dependent protein kinase in the nucleus. This study further shows that the expression vectors encoding C2/CREB and NLSCα are valuable tools for the investigation of CREB-mediated gene transcription and the biological functions of CREB. CREB is a key molecule in neuronal survival, as demonstrated by the fact that a dominant-negative A-CREB induced apoptosis in sympathetic neurons grown in NGF [34]. The C2/CREB fusion protein, that directs CREB-mediated gene transcription in the absence of PKA activation, can be used in gain-of-function experiments to investigate the cytoprotective activity of CREB on the molecular level. Most importantly, the C2/CREB protein can be used to identify anti-apoptotic genes that are controlled by CREB. Likewise, expression of NLSCα will permit the analysis of PKA-regulated gene transcription, without influencing other functions of PKA in the cytosol.

## Methods

### Reporter constructs

The glucose-6-phosphatase promoter/luciferase reporter genes pG6PCRE1/CRE2luc, pG6PCRE1mut/CRE2luc, and pG6PCRE1/CRE2mutluc were constructed by the insertion of the annealed oligonucleotides depicted in Fig. 1B with KpnI/XhoI cohesive ends into the KpnI/XhoI sites of plasmid pHIVTATAluc [35]. The glucose-6-phosphatase promoter/luciferase reporter genes pG6PCRE1luc and pG6PCRE2luc that contain either the CRE1 or the CRE2 sequence derived from the human glucose-6-phosphatase gene were constructed by the insertion of the annealed oligonucleotides depicted in Fig. 1C with XhoI/SalI cohesive ends into the XhoI/SalI sites of plasmid pHIVTATA-CAT [36]. These plasmids termed pG6PCRE1CAT and pG6PCRE2CAT were digested with XbaI, filled in with the Klenow fragment of DNA polymerase I, recut with XhoI and cloned into plasmid pGL3-Basic (Promega). The reporter gene pTNFα(CRE/AP-1)^2^luc, that contains two copies of the CRE/AP1 element of the human tumor necrosis factor α gene, was generated in a similar way with annealed oligonucleotides encompassing the sequence depicted in Fig. 1D. Plasmid pUAS^5^luc containing the luciferase reporter gene, a TATA box derived from the HIV long terminal repeat, an initiator element from the adenovirus major late promoter, and five binding sites for GAL4 (termed 'upstream activating sequence', UAS) has been described [35].

### Expression vectors

The expression vector pCMV-FLAG-C2/CREB, encoding a constitutively active CREB2/CREB chimera, has been described [26]. The CREB2/CREB fusion protein consists of the amino-terminal 187 amino acids from CREB2, encompassing the phosphorylation-independent transcriptional activation domain, and amino acids 182 to 326 of CREB, including the bZIP domain. Expression vectors encoding CREB, CREBS133A, and K-CREB were kind gifts of Wilhart Knepel and Elke Oetjen, Department of Molecular Pharmacology, University of Göttingen, Germany. The expression vector encoding a constitutively active CREB2/ATF2 fusion protein termed C2/ATF2 has been described [27]. The GAL4 expression plasmid pFA2CREB was purchased from Stratagene. The fusion protein encodes the transcriptional activation sequence of CREB (amino acids 1–281) fused to the DNA-binding domain of GAL4. An expression vector encoding the catalytic subunit of PKA (pCMVCα) was a kind gift of Michael Uhler from the University of Michigan, Ann Arbor [37]. The construction of the NLSCα encoding expression vector has been described [26]. NLSCα has a triple FLAG-tag and a nuclear localization signal on the N-terminus, followed by amino acids 19 to 351 of Cα. The expression vector pRSVβ, encoding β-galactosidase under the control of the Rous sarcoma virus long terminal repeat, has been described [29].

### Cell culture, transfections, and reporter gene assays

Human HepG2 hepatoma cells were cultured and transfected as described [38]. The amounts of expression vectors transfected are indicated in the figure legends. The luciferase reporter plasmids (1 μg) and the internal reference plasmid pRSVβ were transfected into cells grown on 60 mm plates. Lysates were prepared forty-eight hours later using cell culture lysis buffer (Promega) and β-galactosidase and luciferase activities were measured as described [35].

### Western Blots

Nuclear extracts were prepared as described [39]. 20 μg of nuclear proteins were separated by SDS-PAGE and the blot was incubated with the M2 monoclonal antibody directed against the FLAG epitope (Sigma, # F3165). Blots were developed with a horseradish peroxidase conjugated anti-mouse secondary antibody and ECL (Amersham, Freiburg, Germany).

## Abbreviations

ATF activating transcription factor

bZIP basic region leucine zipper

CRE cAMP response element

CREB cAMP response element binding protein

G6P glucose-6-phosphatase

PKA protein kinase A

TNF tumor necrosis factor

## Authors' contributions

GT designed the study, generated the reporter plasmids and the expression vectors encoding C2/CREB, C2/ATF2, and NLSCα, performed part of the transfection experiments and drafted the manuscript. JAS performed the transfection experiments depicted in Figs. 3, 4C, and 5. LS performed the C2/ATF2 transfection experiments depicted in Figs. 6. All authors read and approved the manuscript.
